# A New Application of Spin and Fluorescence Double-Sensor Molecules

**DOI:** 10.3390/molecules28072978

**Published:** 2023-03-27

**Authors:** Flórián Bencze, Balázs Bognár, Tamás Kálai, László Kollár, Zoltán Nagymihály, Sandor Kunsági-Máté

**Affiliations:** 1 Department of Organic and Medicinal Chemistry, Faculty of Pharmacy, University of Pécs, Honvéd Street 1, H-7624 Pécs, Hungary; 2 János Szentágothai Research Center, University of Pécs, Ifjúság útja 20, H-7624 Pécs, Hungary; 3 ELKH-PTE Research Group for Selective Syntheses, Ifjúság útja 6, H-7624 Pécs, Hungary; 4 Department of Physical Chemistry and Materials Science, Faculty of Sciences, University of Pécs, Ifjúság 6, H-7624 Pécs, Hungary

**Keywords:** free radical, fluorescence, sensor molecule, molecular interactions, cavitand, fluorescence polarization

## Abstract

EPR imaging techniques are known to be successful tools for mapping living bodies, especially because of the high transparency of tissues in the microwave range. This technique assumes the presence of radicals whose in vivo transport is also controlled by serum albumins. Accordingly, in this study, the interactions between 3-hydroxymethyl-1-oxyl-4-(pyren-1-yl)-2,2,5,5-tetramethyl-2,5-dihydro-1*H*-pyrrole radical and the human serum albumin molecules were investigated. To clarify the adsorption processes of this radical onto the surface of human serum albumin (HSA), the interaction of the OMe derivative of the radical was also examined parallel with the studies on the radical—HSA interactions. Considering the solubility issues and also to modulate the transport, inclusion complexes of the radical with a cavitand derivative were also studied. The latter interactions were observed through fluorescence spectroscopy, fluorescence polarization, and by EPR spectroscopy. As a double-sensor molecule, we found that the fluorophore nitroxide is a good candidate as it gave further information about host-guest interactions (fluorescence, fluorescence polarization, and EPR). We also found that in the presence of a cavitand, a complex with greater stability was formed between the sensor molecule and the human serum albumin. Based on these observations, we can conclude that applying this double-sensor (spin, fluorescent) molecule is useful in cases when different interactions can affect the EPR measurements.

## 1. Introduction

**Figure 1:**
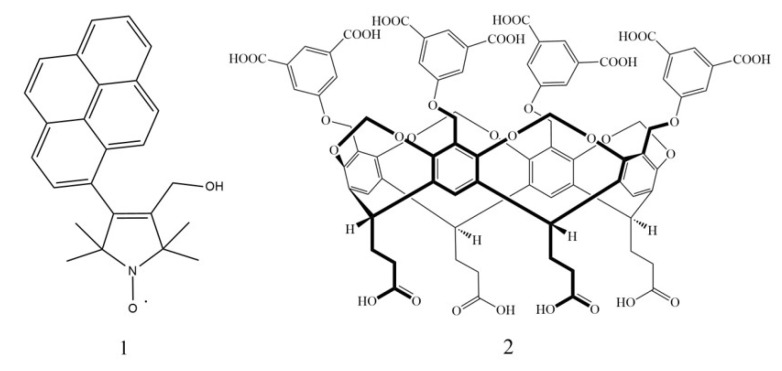
The chemical structure of the model fluorescent 3-hydroxymethyl-1-oxyl-4-(pyren-1-yl)-2,2,5,5-tetramethyl-2,5-dihydro-1*H*-pyrrole (**1**, NO, **left**) and the macrocyclic tetrakis(3,5-dicarboxylatophenoxy)-cavitand molecules (**2**, TDC, **right**).

**Figure 2:**
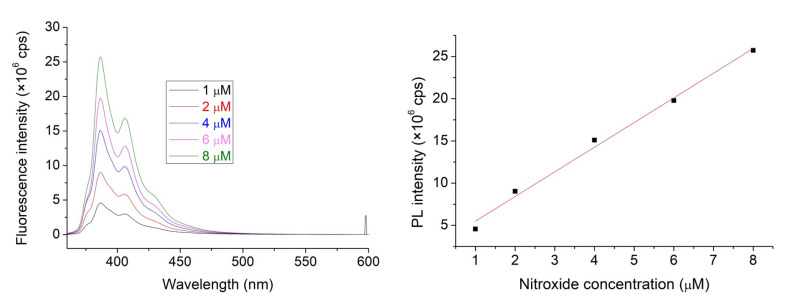
The emission spectra of NO with increasing concentration within the 1–100 μM range (**left**). Linear correspondence of emission to the fluorophore nitroxides concentration (adj. R^2^ = 0.990, **right**).

Fluorescent probes and sensors have been introduced to safely and diversely examine biological systems [1,2] as one of their many qualities. Fluorescent probes and sensor molecules can be more easily modified and used than their isotopic counterparts [3], one of which is double-sensor molecules. By introducing other moieties, such as unpaired electron-containing ones, the methods of examining their interactions can be vastly expanded. Nitroxide free radicals are among the more stable radicals used for creating probes containing fluorescent moieties. These reagents can be used in analytical chemistry to detect reactive oxygen species (ROS) [4] by electron spin resonance spectroscopy (EPR-spectroscopy) and by fluorescence spectroscopy [5,6], or fluorophore-attached nitroxides can be utilized as redox indicators [7]. It has also been documented how these nitroxides interact with certain proteins, such as BSA (Bovine Serum Albumin) [8], offering one more analytical tool. Fluorophores as light-sensitive molecules have been in the limelight of clinical research as well since many are used as antibacterial [9], antifungal [10], and chemotherapeutic agents [11]. Since pyrene is widely considered a useful compound for determining solvent environments, 3-hydroxymethyl-1-oxyl-4-(pyren-1-yl)-2,2,5,5-tetramethyl-2,5-dihydro-1*H*-pyrrole radical [12] (Figure 1, **1**) was used in this study as a model molecule for a pharmaceutical agent in a system containing human serum albumin (HSA). This study focuses on the nitroxide **1** interaction with HSA and with the tetrakis(3,5-dicarboxylatophenoxy)-cavitand [13] (Figure 1, **2**). Within this model study, the **2** was applied as an agent that could change the rate and stability of complex formation between **1** and the HSA molecules. This model has been investigated by fluorescence, fluorescence polarization, and EPR spectroscopy [14,15,16]. In our previous publications, we presented several possibilities for examining weak molecular interactions with fluorescence or fluorescence polarization measurements. In our present research, we aimed to expand the possibilities of examining these interactions by using dual sensors. Since biological systems are transparent at the wavelength used in EPR spectroscopic studies, nitroxide–fluorophore sensors provide an additional opportunity to understand the biological role of weak interactions.

## 2. Results and Discussion

### 2.1. Fluorescence Studies

Considering the limited solubility of **1** in water, experiments have been performed in a mixture of acetonitrile (ACN) and water, composed 1:9 ratio. This solvent was used to prepare the **1**, **2**, and HSA stock solutions. Figure 2 shows the straight linear dependence of PL intensities of nitroxide **1** on its concentration. An excitation wavelength of 347.0 nm was selected for this study since neither the HSA nor the cavitand **2** show considerable emission under excitation at this wavelength.

To determine the complex stabilities associated with the interaction between nitroxide **1** and HSA, the emission spectra of 1 μM nitroxide **1** in the absence and in the presence of HSA with increasing concentrations were recorded. To determine the effect of the presence of cavitand **2** on the interaction above, the experiments were repeated in the presence of 9.375 μM cavitand **2**. Emission peaks observed at 387.5 nm have been used later for data evaluation.

**Figure 3:**
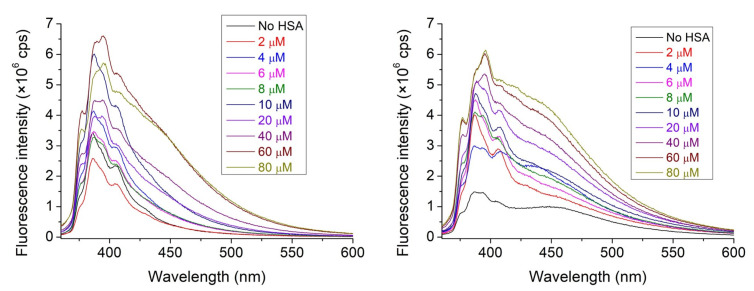
The fluorescence spectra of nitroxide **1** (1 μM) in the absence and in the presence of HSA with increasing concentrations. Solutions in the absence (**left**) and solutions in the presence (**right**) of cavitand **2** were recorded. The concentration of cavitand **2** was set to 9.375 μM.

The emission intensity of nitroxide **1** always increased in the presence of HSA. Considering the known quenching property of water molecules on the emission of any fluorophore, this property reflects at least partial destruction of the hydration shell of nitroxide **1** during adsorption onto the surface of the protein. A slight increase (about 25%) in intensity is observed in the presence of cavitand **2** in the HSA-free solutions (Figure 3), highlighting that the cavitand includes the nitroxide **1** derivative with its fluorescent pyrene moiety.

**Figure 4:**
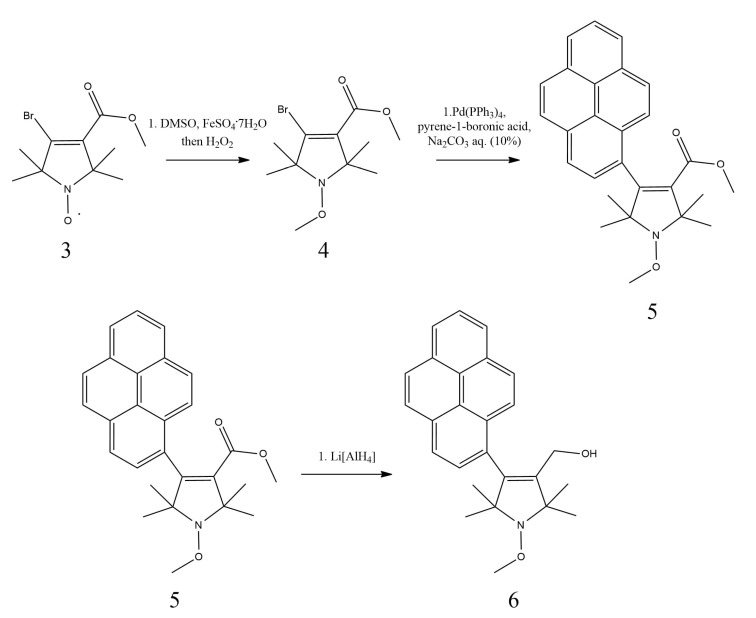
Synthesis route of the OMe derivative compound **6**.

However, this anomaly also introduces the probability of the nitroxide unpaired electron playing a role, as the molecule has the potential to undergo a reduction reaction that may further amplify the observed intensity. To fully comprehend this effect and its exclusion, we created the *O*-methyl derivative of our probe molecule **1** (Figure 4).

#### The Synthesis of the Methyl Derivative of Compound **1** (**6**)

The desired compound **6** was synthesized in a three-step process. The Fenton reaction of compound **3** [17] gave compound **4** via methyl radical generation from DMSO, which gave compound **5** through a Suzuki coupling with pyrene-1-boronic acid. The desired **6** alcohol was achieved by the reduction of the ester **5** with LiAlH_4_.

**Figure 5:**
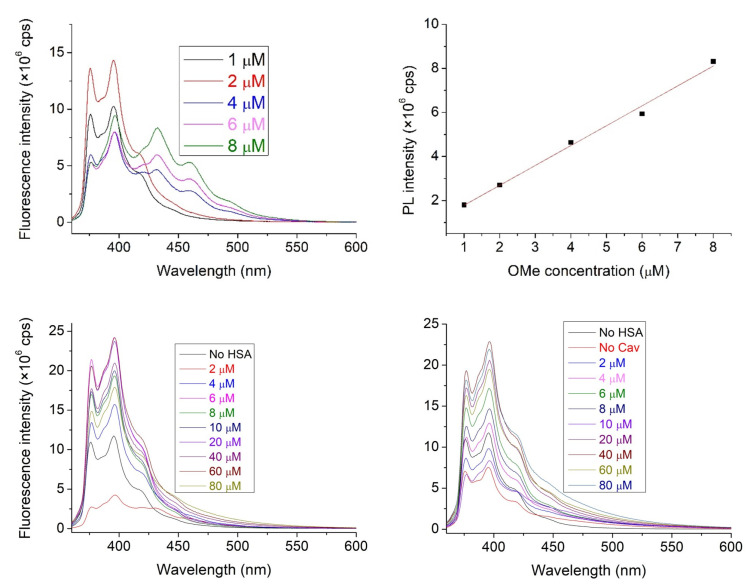
The emission spectra of **6** with an increasing concentration within the 1–100 μM range (**top left**) and linear correspondence of emission to the fluorophore OMe concentration (adj. R^2^ = 0.994, **top right**). The fluorescence spectra of compound **6** (1 μM) in the absence and the presence of HSA with increasing concentrations. Solutions in the absence (**bottom left**) and solutions in the presence (**bottom right**) of cavitand **2** were recorded. The concentration of cavitand **2** was set to 9.375 μM.

We have made the preliminary examinations in the same manner as with nitroxide. The excitation wavelength had to be changed from 347.0 nm to 341.0 nm. Just as before, neither HSA nor cavitand **2** had shown considerable emission at this wavelength; however, ACN and water were more visible than in the previous experiment. Moreover, the emission peaks that were used for evaluation were changed to 433.0 nm based on the spectra of **6** (Figure 5).

The increased intensity is notable in the solutions containing either HSA or cavitand **2**, as this effect can be attributed to the nitroxide quenching effect not being present. Although it does not answer definitively to the supposed reaction of the nitroxide, it gives the pretense that this side reaction is not prevalent.

**Figure 6:**
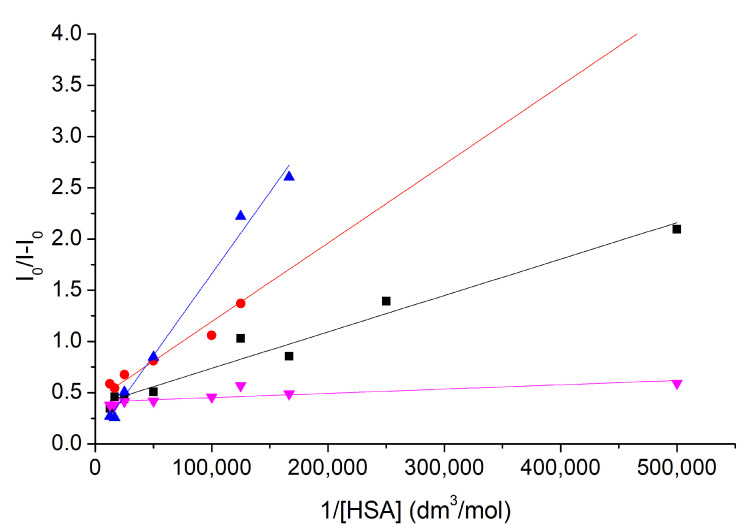
The Benesi–Hildebrand plots to determine the complex stabilities of nitroxide **1** and compound **6** with HSA in the absence (blue and red) and in the presence (purple and black) of cavitand **2**.

Via the Benesi–Hildebrand method, the complex stabilities of nitroxide **1** and compound **6** with HSA in the absence and in the presence of cavitand **2** have been calculated. Results reflect the formation of stable 1: HSA and **6**: HSA complexes. Figure 6 and Table 1 summarize these results.

### 2.2. Measuring the Complex Stabilities by Fluorescence Polarization

**Figure 7:**
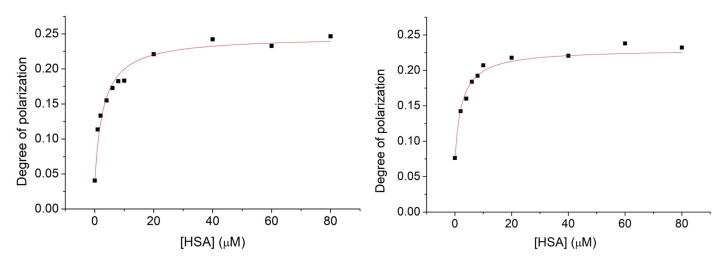
Fluorescence polarization spectra of nitroxide **1** (1 μM) in the presence of HSA with increasing concentration within the range of 0–80 μM. Experiments were performed in the absence (**left**) and in the presence of cavitand **2** (**right**).

The methods based on the change of PL intensities are associated with several uncertain aspects. One example is that the decrease in the PL intensity is probably induced by the interaction between the aromatic moiety of nitroxide **1** with the π-faces of the protein, which competes with the effect of removal of water molecules from the hydration shell of **1**. As a result of that, the PL intensities are increased. The dominant impact is the latter, while the other disturbs the measurements. To avoid any discrepancies, the fluorescence polarization of nitroxide **1** in the model applied above has been measured (Figure 7). Similarly, as in the PL intensity studies, the concentration of nitroxide **1** was kept at 1 μM, while the concentration of HSA was varied from 0 μM up to 20 μM. Measurements were performed either in the absence or in the presence of 9.375 μM cavitand **2**. To clarify the possible effect on the nitroxide reduction to hydroxylamine by the HSA, the measurements above have been repeated with compound **6**.

The degree of polarization shows elevated values with increasing HSA concentration. This property highlights hindered rotation of the radical in the presence of HSA, suggesting considerable adsorption of nitroxide **1** onto the HSA surface. In both cases, when the nitroxide **1**—HSA complex formed in the absence or in the presence of the cavitand **2**, saturated solutions can be obtained above the 40 μM HSA concentration. The fluorescence polarization spectra were evaluated quantitatively by fitting Equation (5) to the data plotted in Figure 7. Stability constants derived either from the fluorescence intensity or degree of polarization data are summarized in Table 1. 

The results based on the fluorescence polarization measurements show higher complex stabilities compared to the stabilities derived from the PL intensities. However, both methods confirm enhanced adsorption of nitroxide **1** onto the surface of HSA in the presence of cavitand **2**. Considering that our preliminary investigation cannot confirm the adsorption of parent cavitand **2** molecules onto the HSA, the enhanced adsorption of **1** in the presence of **2** is probably due to the reduced Coulomb repulsion between deprotonated OH group of **1** radical and the HSA by inclusion the charged moiety of the radical by the cavitand **2**.

**Figure 8:**
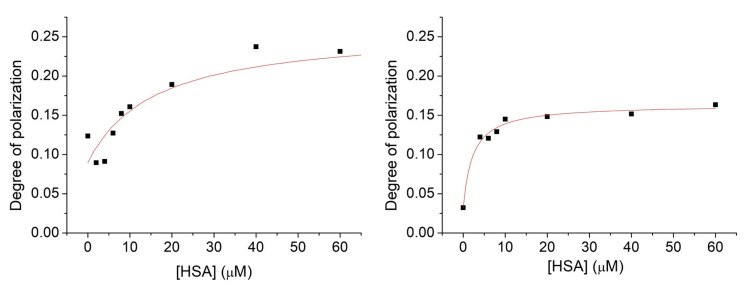
Fluorescence polarization spectra of compound **6** (1 μM) in the presence of HSA with an increasing concentration within the range of 0–80 μM. Experiments were performed in the absence (**left**) and in the presence of cavitand **2** (**right**).

For comparison, we have measured the fluorescent polarization of compound **6** in the absence and presence of cavitand **2** with increasing concentrations of HSA. Spectra were evaluated quantitatively by the same fitting method as before (Figure 8).

Results derived either by the fluorescence or the polarization measurements highlight at least an order of magnitude difference in the stability constant of **1** upon the introduction of cavitand into the system. Much lower differences in the stabilities derived by the fluorescence intensity or polarization studies were obtained in complex formations acting by compound **6**. This could be attributed to the effects of the nitroxide reduction to hydroxylamine by the HSA. The slight enhancement in the interactions of **6** with HSA shown by both the PL intensity and polarization measurements is probably due to the slight electron-releasing property of the methyl group. The uncharged OMe moiety of **6** itself results in weaker Coulomb repulsion toward the electron-rich binding site of the HSA compared to the case of the **1**—HSA interactions. This behavior is also supported by the fact that the presence of the cavitand **2** does not improve the stability of the **6**—HSA complexes with the same content as in the case of the formation of the **1**—HSA complexes. 

### 2.3. EPR Measurements

**Figure 9:**
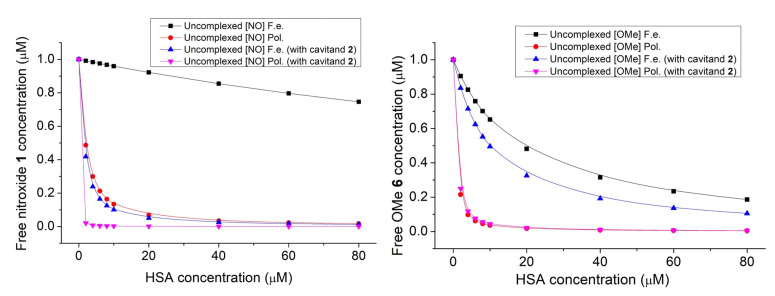
The effect of presence of HSA and cavitand **2** on the free nitroxide **1** (**left**) and OMe derivative **6** (**right**) fluorophore concentration.

**Figure 10:**
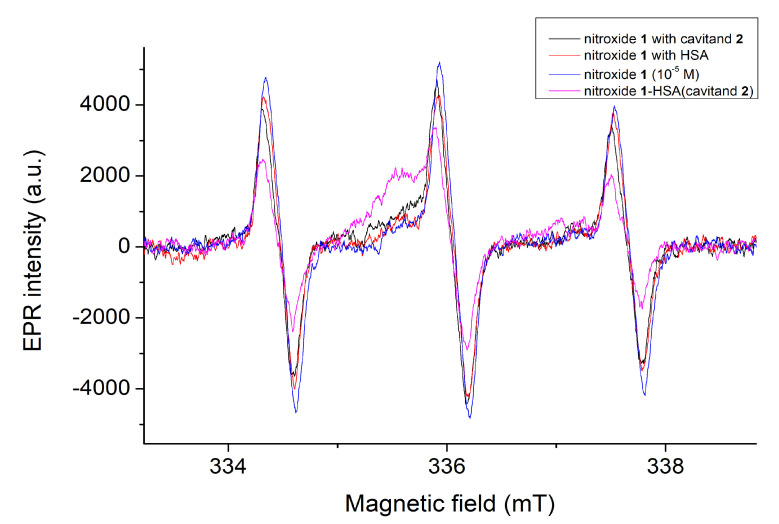
The EPR spectra of nitroxide **1** in the absence and in the presence of HSA and/or cavitand **2**. Distortion on the middle peaks in the presence of HSA and/or cavitand **2** confirms the complex formation.

Due to the differences between the complex stabilities derived from the PL intensities and from the fluorescence polarization, the interaction was also examined by EPR spectroscopy. To set the appropriate composition of samples for EPR studies, the concentration of free nitroxide **1** was calculated from the stability constants summarized in Table 1 as a function of the concentration of HSA. Using Figure 9, the following four solutions have been prepared for EPR studies: sample 1: nitroxide **1** (10 μM); sample 2: nitroxide **1** (10 μM) + cavitand **2** (10 μM); sample 3: nitroxide **1** (10 μM) + HSA (100 μM); sample 4: nitroxide **1** (10 μM) + cavitand **2** (10 μM) + HSA (100 μM). Results are summarized in Figure 10.

The concentration of the unbound nitroxide fluorophore can be monitored and calculated on the basis of integrated peaks. This area is in linear relation with the concentration of the free nitroxide **1** radical. In agreement with the fluorescence and fluorescence polarization studies, the concentration of the free nitroxide molecules reduces slightly (with 16%) when only the HSA molecules are present in the samples. In contrast, the concentration of nitroxide **1** reduces by about 38% in cases when both the HSA and cavitand **2** are present in the samples. In this instance, the EPR spectra prove that some of the nitroxide reduces to hydroxylamine, giving a greater signal intensity reduction. Further examinations are planned to clarify this property at the molecular level.

## 3. Materials and Methods

### 3.1. Materials

The solvent was a 1:9 mixture of ACN and distilled water solution [18] and was used in every experiment as a solvent. The synthesis of 3-hydroxymethyl-1-oxyl-4-(pyren-1-yl)-2,2,5,5-tetramethyl-2,5-dihydro-1*H*-pyrrole radical and tetrakis(3,5-dicarboxylatophenoxy)-cavitand was carried out in our institute according to published methods [12,13], and their ^1^H NMR scan was taken. The applied solvent (acetonitrile) and human serum albumin was purchased from Merck (Darmstadt, Germany).

**3-Hydroxymethyl-1-oxyl-4-(pyren-1-yl)-2,2,5,5-tetramethyl-2,5-dihydro-1*H*-pyrrole Radical (1):**

^1^H NMR (500.1 MHz, DMSO-d_6_ + (PhNH)_2_): δ 0.995 (s, 3H, -CH_3_), 1.376 (s, 3H, -CH_3_), 1.474 (s, 3H, -CH_3_), 1.551 (s, 3H, -CH_3_), 3.725 (d, *J* = 9.8 Hz, 1H), 3.838 (d, *J* = 10.0 Hz, 1H), 8.185 (m, 9H, Ar-H)

**Tetrakis(3,5-dicarboxylatophenoxy)-cavitand (2):**

^1^H NMR (500.1 MHz, DMSO-d_6_): δ 2.32 (t, *J* = 6.6 Hz, 8H), 2.73 (tt, *J* = 6.6 Hz, 8H), 4.47 (d, *J* = 7.6 Hz, 4H, inner of OCH_2_O), 4.77 (q, 4H, –CH_2_–CH), 4.92 (s, 8H, Ar–CH_2_–), 5.89 (d, *J* = 7.6 Hz, 4H, outer of OCH_2_O), 7.68 (s, 8H, Ar–H), 7.84 (s, 4H, Ar–H). 8.07 (s, 4H, Ar–H), 10.17 (s, 4H, COOH), 12.89 (s, 8H, COOH).

#### Synthesis Route of the Methyl Derivative of the Fluorophore

The synthesis of the methyl derivative has taken several steps (Figure 4), and the starting compound has already been documented [17]. Unidentified peaks are visible in NMR, presumably from hexane.

**Methyl 4-bromo-1-methoxy-2,2,5,5-tetramethyl-2,5-dihydro-1*H*-pyrrole-3-carboxylate (4):**

First, 30% aq. H_2_O_2_ (8 mL) was added dropwise to a stirred solution of compound **3** (1.39 g, 5.0 mmol) and FeSO_4_∙7H_2_O (6.90 g, 25.0 mmol) in DMSO (30 mL) and ACN (15mL) at 0 °C, in 10-min 3–5 drop intervals cautiously. After the starting material (TLC monitoring) was consumed, the reaction mixture was diluted with water (50 mL). The aqueous solution was extracted with Et_2_O (3 × 30 mL). The organic phase was dried (MgSO_4_), filtered, and evaporated. The crude product was purified by flash column chromatography (Hexane/Et_2_O 2:1) to give a colorless oil (1,20 g, 82%). TLC R_f_ = 0.50 (Hexane/Et_2_O 5:1); MS (EI) m/z (% relative intensity) = 293 (M^+^, 2.36), 291 (2.34), 278 (43.6), 276 (45.0), 197 (100), 166 (28.1), 134 (13.6); IR $\overset{-}{\nu }$ = 2980, 2937, 1717 (C=O), 1617 cm^−1^; ^1^H NMR (CDCl_3_): δ 1.35 (s, 6C, -CH_3_), 1.43 (s, 6H, -CH_3_), 3.72 (s, 3H, -OCH_3_), 3.82 (s, 3H, -OCH_3_); ^13^C NMR (CDCl_3_): δ 21.9, 29.1, 51.6, 65.4, 70.8, 72.0, 134.5, 163.7. Anal. calcd. for C_11_H_18_BrNO_3_: C, 45.22; H, 6.21; N, 4.79%; found: C, 45.19; H, 6.19; N, 4.81%.

**Methyl 1-methoxy-2,2,5,5-tetramethyl-4-(pyren-1-yl)-2,5-dihydro-1*H*-pyrrole-3-carboxylate (5):**

A solution of compound **4** (582 mg, 2.0 mmol) in dioxane (15 mL) was purged with N_2_ stream for 5 min., then Pd(PPh_3_)_4_ (100 mg, 0.1 mmol) and pyrene-1-boronic acid (492 mg, 2.0 mmol) and then the 10% aq. Na_2_CO_3_ (10 mL) was added, and the mixture was stirred and refluxed under N_2_ for 2 h. After cooling, the dioxane was evaporated; water (10 mL) was added, and the aqueous phase was extracted with EtOAc (2 × 20 mL). The organic phase was dried (MgSO_4_), filtered, evaporated, and after flash column chromatographic purification (Hexane/Et2O), compound **5** was obtained as a white/pale yellow solid (490 mg, 59%). Mp. 179–180 °C; TLC R_f_ = 0.55 (Hexane/Et_2_O 4:1); MS (EI) *m*/*z* (% relative intensity) = 413 (M^+^, 12.4), 398 (100), 367 (30.8), 338 (9.50), 308 (17.7), 199 (8.30); IR $\overset{-}{\nu }$ = 3041, 2982, 2962, 2924, 1701 (C=O), 852 cm^−1^; ^1^H NMR (CDCl_3_): δ 1.30 (s, 3C, -CH_3_), 1.51 (s, 3H, -CH_3_), 1.66 (s, 3H, -CH_3_), 1.79 (s, 3H, -CH_3_), 3.20 (s, 3H, -OCH_3_), 3.86 (s, 3H, -OCH_3_), 7.78 (s, 2H, Ar-H), 8,04 (t, *J* = 7.5 Hz, 1H, Ar-H), 8.09–8.23 (m, 6H, Ar-H); ^13^C NMR (CDCl_3_): δ 21.4, 28.4, 50.9, 65.5, 69.8, 72.8, 124.0, 124.5, 124.6, 124.8, 125.0, 125.2, 126.0, 127.4, 127.5, 129.2, 130.6, 131.0, 131.4, 135.3, 164.7. Anal. calcd. for C_27_H_27_NO_3_: C, 78.42; H, 6.58; N, 3.39%; found: C, 78.40; H, 6.55; N, 3.40%.

**(1-methoxy-2,2,5,5-tetramethyl-4-(pyren-1-yl)-2,5-dihydro-1*H*-pyrrol-3-yl)methanol (6):**

To a stirred solution of compound **5** (450 mg, 1.1 mmol) in Et2O (10 mL), LiAlH_4_ (114 mg, 3.0 mmol) was added under N_2_. The solution was stirred at room temperature until the complete consumption of the starting material (TLC monitoring); then, the solution was poured onto 10% aq. NaOH solution and crushed ice; then, it was diluted with Et_2_O (20 mL). The organic phase was separated, and the aqueous phase was extracted with CHCl_3_ (2 × 20 mL). The combined organic phase was dried (MgSO_4_), filtered, evaporated, and after flash column chromatography, compound **6** was obtained as a white solid (330 mg, 77%). Mp. 139–140 °C; TLC R_f_ = 0.35 (Hexane/Et_2_O 1:1); MS (EI) *m*/*z* (% relative intensity) = 385 (M^+^, 8.00), 370 (100), 339 (14.4), 309 (27.8); IR $\overset{-}{\nu }$ = 3301, 3044, 2924, 1600, 853 cm^−1^; ^1^H NMR (CDCl_3_): δ 1.31 (s, 6C, -CH_3_), 1.57 (s, 3H, -CH_3_), 1.68 (s, 3H, -CH_3_), 3.87 (s, 3H, -OCH_3_), 3.94 (s, 2H, -CH_2_-), 7.84 (d, *J* = 8.0 Hz, 1H, Ar-H), 8.06 (t, *J* = 7.5 Hz, 1H, Ar-H), 8.10–8.25 (m, 7H, Ar-H); ^13^C NMR (CDCl_3_): δ 22.5, 29.5, 57.9, 65.4, 69.9, 71.7, 124.3, 124.8, 124.9, 125.2, 125.4, 126.2, 127.4, 127.6, 127.9, 130.1, 130.2, 130.8, 131.0, 131.4, 141.3, 141.9. Anal. calcd. for C_26_H_27_NO_2_: C, 81.01; H, 7.06; N, 3.63%; found: C, 81.04; H, 7.05; N, 3.64%.

### 3.2. Methods

Fluorimetric measurements were performed with a Fluorolog τ3 spectrofluorometer (Jobin-Yvon/SPEX, Longjumeau, France). Fluorescence spectra were recorded using 347.0 and 341.0 nm excitation wavelengths to NO and OMe compounds, respectively. The emission values obtained at 387.5 and 433.0 nm were used for data evaluation. For data collection, the photon counting method with 0.1 s integration time was used. Excitation and emission bandwidths were set to 5 nm. To avoid the inner filter effect, a 2 mm thickness of the fluorescent probes with right-angle detection was applied.

To determine the thermodynamic parameters associated with the complexation reaction of the NO and HSA both in the absence and presence of TDC, samples with a constant concentration of NO (1 µM) and TDC (9.375 μM) with different concentrations of HSA (0–100 µM) were prepared in ACN:distilled water (1:9) solution and measured immediately using 347.0 nm excitation wavelength at 298.15 K.

A MiniScope MS 200 (Magnettech GmbH, Berlin, Germany) spectroscope was utilized to detect the produced free radicals and to examine the NO’s complexation. The amplitude of the EPR signal is proportional to the number of unpaired electrons present in the sample, facilitating the quantification of free radicals [19]. The amplitudes were determined using the MiniScopeCtrl software. The following EPR settings were applied for all experiments: B0-field: 335.9723 mT, range: 10.0485 mT, sweep time: 30.0 s, modulation: 0.300 mT, and microwave attenuation: 15.0 dB. All measurements were carried out at room temperature (298 K).

The mass spectra were recorded with a GCMS-2020 (Shimadzu, Tokyo, Japan) operated in EI mode (70 eV) and a ThermoScientific Q-Extractive HPLC/MS/MS with ESI(+) ionization (Thermo Scientific, Waltham, MA, USA). ^1^H-NMR spectra were recorded with Bruker Avance 3 Ascend 500 system (Bruker, BioSpin Corp., Karlsruhe, Germany) operated at 500 MHz, and ^13^C-NMR spectra were obtained at 125 MHz in CDCl_3_ at 298 K. IR spectra were obtained with a Bruker Alpha FT-IR instrument (Bruker Optics, Ettlingen, Germany) with ATR support on a diamond plate. All spectra are shown in the Supplementary Material, Figures S1–S3.

#### Data Evaluation

Stability constants (*K*, dm^3^/mol) of NO–HSA complexes were calculated using the Benesi–Hildebrand equation, assuming 1:1 complex stoichiometry:

(1) $$\frac{I_{0}}{I-I_{0}}=\frac{1}{\left[HSA\right]\cdot \left[NO\right]\cdot K}+\frac{1}{\left[NO\right]}$$

where *I*_0_ and *I* are the fluorescence emission intensities of *NO* in the absence and in the presence of the host, respectively; [*HSA*] is the molar concentration of the host molecule, while [*NO*] is a constant.

The degree of fluorescence polarization, which was calculated as

(2) $$P=\frac{\left(I_{VV}-G\times I_{VH}\right)}{\left(I_{VV}+G\times I_{VH}\right)}$$

where *I_VV_* and *I_VH_* are intensities of vertically and horizontally polarized emissions, respectively, and *G* is the measured instrument factor. Fluorescence polarization values depend on the rotational freedom of the excited molecules (NO in our experiments), therefore directly mirroring microenvironment-related molecular motions such as binding of the tested fluorophore to a macromolecule. For calculating the degree of polarization, 100 measuring points were averaged.

The polarization-based approach was performed, and the binding parameters were determined similarly as had been described earlier by Poór et al. [20]:

(3) $$\alpha =\frac{\left(P-P_{f}\right)}{\left[\theta \times \left(P-P_{b}\right)+P-P_{f}\right]}$$

where *α* is the bound fraction of the toxin, *P* is the measured polarization, and *P_f_* and *P_b_* are fluorescence polarization values of free and bound NO, respectively. Polarization value of the free nitroxide fluorophore (*P_f_*) in ACN:water was determined when only 1 μM NO was present in the solution without albumin. Additionally, the *P_b_* value was measured using 1 μM NO and 60 μM HSA (HSA being above the saturating concentration).

Furthermore:

(4) $$\theta =\frac{\varepsilon_{b}\times \Phi_{b}}{\varepsilon_{f}\times \Phi_{f}}$$

where *ε_b_* and *ε_f_* are the molar absorptivities of bound and free toxin, *Φ_b_* and *Φ_f_* are the fluorescent quantum yields of the bound and free NO. Using Equation (2), the polarization can be expressed as a function of *α*, *P_f_*, *P_b_* and *θ*:

(5) $$P=\frac{P_{f}\left(\alpha -1\right)-\alpha \theta P_{b}}{\alpha -\alpha \theta -1}$$

The bound fraction of the nitroxide fluorophore, *α* can be expressed as the concentration of the bound nitroxide fluorophore ([*NO*:*HSA*]) divided by the total *NO* concentration ([*NO*]^0^):

(6) $$\alpha =\frac{\left[NO:HSA\right]}{{\left[NO\right]}^{0}}$$

However, assuming the 1:1 stoichiometry of the *NO*:*HSA* complex, the concentration of the NO:HSA complex can be written as the function of the total concentration of NO ([*NO*]^0^) and HSA ([*HSA*]^0^) and the stability constant (*K*) of the complex formation:

(7) $$\left[NO:HSA\right]=\frac{\left({\left[NO\right]}^{0}+{\left[HSA\right]}^{0}+\frac{1}{K}\right)\pm \sqrt{{\left({\left[NO\right]}^{0}+{\left[HSA\right]}^{0}+\frac{1}{K}\right)}^{2}-4{\left[NO\right]}^{0}{\left[HSA\right]}^{0}}}{2}$$

Inserting Equation (7) into the expression of *α* in Equation (6), then inserting into Equation (5), the polarization of the system can be expressed as a function of HSA concentration. Therefore, the stability constant of the NO–HSA interaction can be determined by nonlinear fitting of Equation (5) to the experimental data.

## 4. Conclusions

In this work, the interactions between 3-hydroxymethyl-1-oxyl-4-(pyren-1-yl)-2,2,5,5-tetramethyl-2,5-dihydro-1*H*-pyrrole radical (**1**) and the human serum albumin molecules have been investigated by fluorescence, fluorescence polarization, and EPR methods. Results confirm the formation of stable complexes of nitroxide **1** with HSA. The presence of a cavitand derivative **2** enhances the complex formation. PL and PL polarization measurements reflect a slight enhancement in the interactions of compound **6** (the diamagnetic derivative of **1**) with HSA. Considering the slight electron-releasing property of the methyl group, the uncharged OMe moiety of **6** itself results in weaker Coulomb repulsion toward the electron-rich binding site of the HSA compared to the case of the **1**—HSA interactions. This behavior is supported also by the fact that the presence of the cavitand **2** does not improve the stability of the **6**—HSA complexes with the same content as in the case of the formation of the **1**—HSA complexes. Considering the broad applications of EPR imaging techniques for mapping living bodies, the model investigated here serves as a test system application of cavitands, improving the sensitivity of EPR imaging in tissues. Results also confirm that the double-sensor probes (spin and fluorescent) can be applied in protein analysis and also in complex systems. With appropriate double-sensor molecules, biological interactions on the molecular scale could be studied in parallel.

## Figures and Tables

**Table 1:** The determined stability constants of nitroxide **1**—HSA and **6**—HSA complexes (assuming 1:1 complexes) derived from fluorescence intensity or degree of polarization data.

Method Applied	logK (1—HSA, Absence of 2)	logK (1—HSA, Presence of 2)
Fluorescence	3.63	5.99
Polarization	5.85	7.66
In case of OMe	logK (**6**—HSA, absence of **2**)	logK (**6**—HSA, presence of **2**)
Fluorescence	4.74	5.03
Polarization	6.48	6.38

## Data Availability

The data presented in this study are available on request from the corresponding author. Samples of the compounds 3-Hydroxymethyl-1-oxyl-4-(pyren-1-yl)-2,2,5,5-tetramethyl-2,5-dihydro-1*H*-pyrrole Radical and Tetrakis(3,5-dicarboxylatophenoxy)-cavitand and **3**–**6** are available from the authors on request in mg scale.

## Supplementary Materials

The following supporting information can be downloaded at: https://www.mdpi.com/article/10.3390/molecules28072978/s1, Figures S1–S3: NMR spectra of compounds **4**, **5** and **6**.
