# Supplementary material for: A New Application of Spin and Fluorescence Double-Sensor Molecules
Source: Molecules. 2023 Mar 27;28(7):2978. doi: 10.3390/molecules28072978 (PMC10096054; doi:10.3390/molecules28072978)
Supplement: Supplementary file 1 [file molecules-28-02978-s001.zip › molecules-2266887-supplementary.pdf]

Supporting Information for

A New Application of Spin and Fluorescence Double-Sensor Molecules

Flórián Bencze [1], Balázs Bognár [1], Tamás Kálai [1,2], László Kollár [2,3], Zoltán Nagymihály [2] and Sandor Kunsági-Máté [1,2,4,*]

[1] Department of Organic and Medicinal Chemistry, Faculty of Pharmacy, University of Pécs, Honvéd Street 1, H-7624 Pécs, Hungary
[2] János Szentágothai Research Center, University of Pécs, Ifjúság útja 20, H-7624 Pécs, Hungary
[3] ELKH-PTE Research Group for Selective Syntheses, Ifjúság útja 6, H-7624 Pécs, Hungary
[4] Department of Physical Chemistry and Materials Science, Faculty of Sciences, University of Pécs, Ifjúság 6, H-7624 Pécs, Hungary
* Correspondence: kunsagi-mate.sandor@gytk.pte.hu

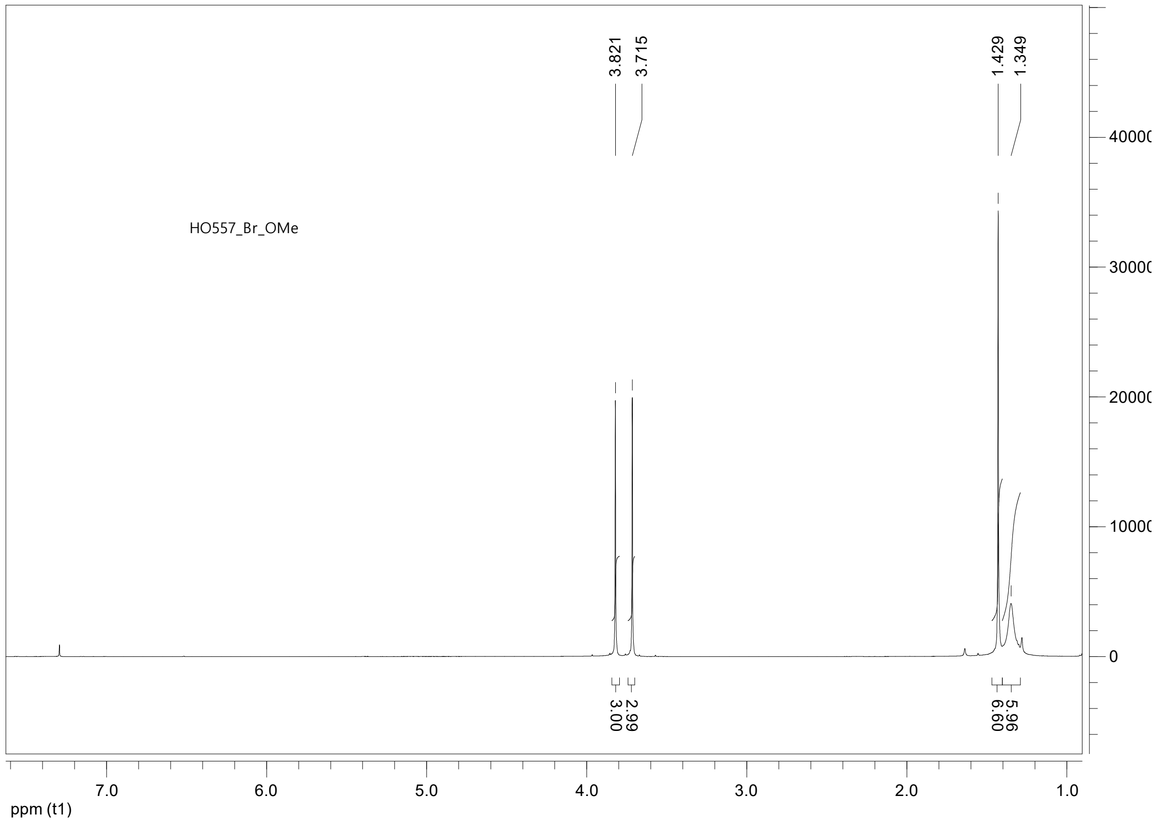

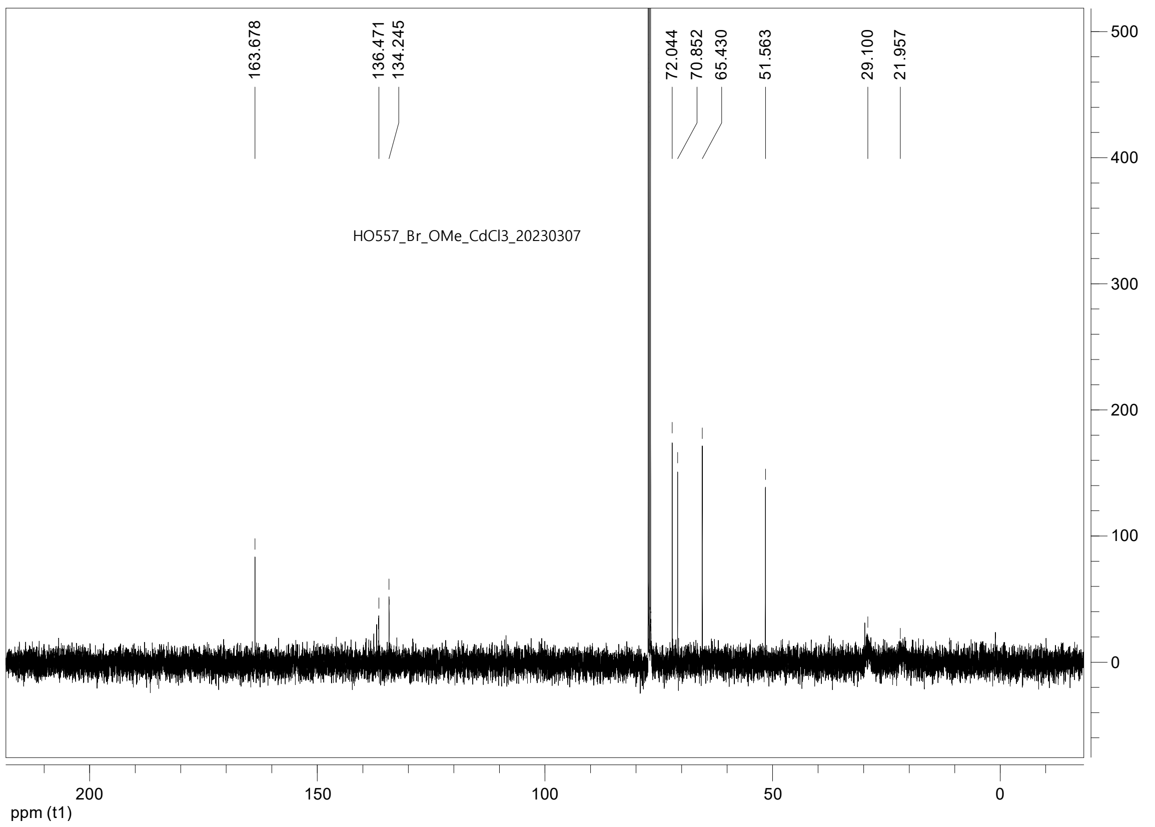

Figure S1: The ^{1}H NMR and ^{13}C NMR of compound **4**.

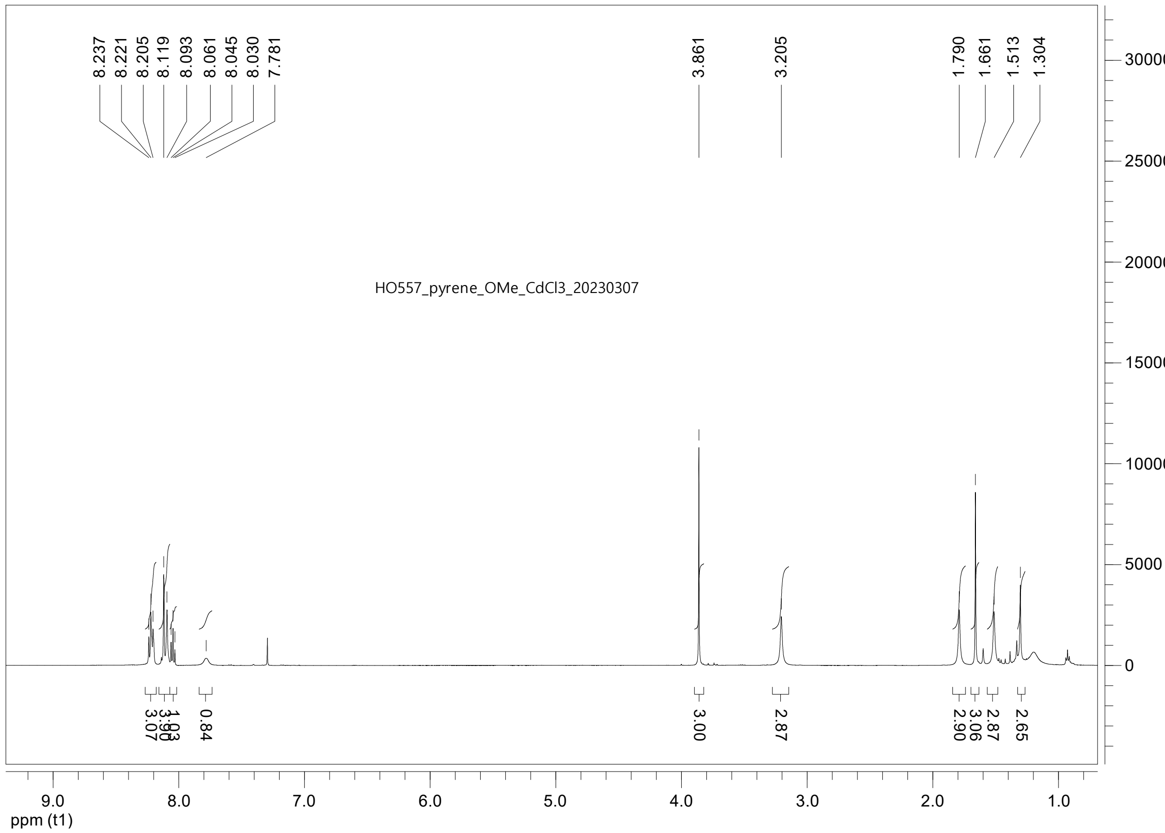

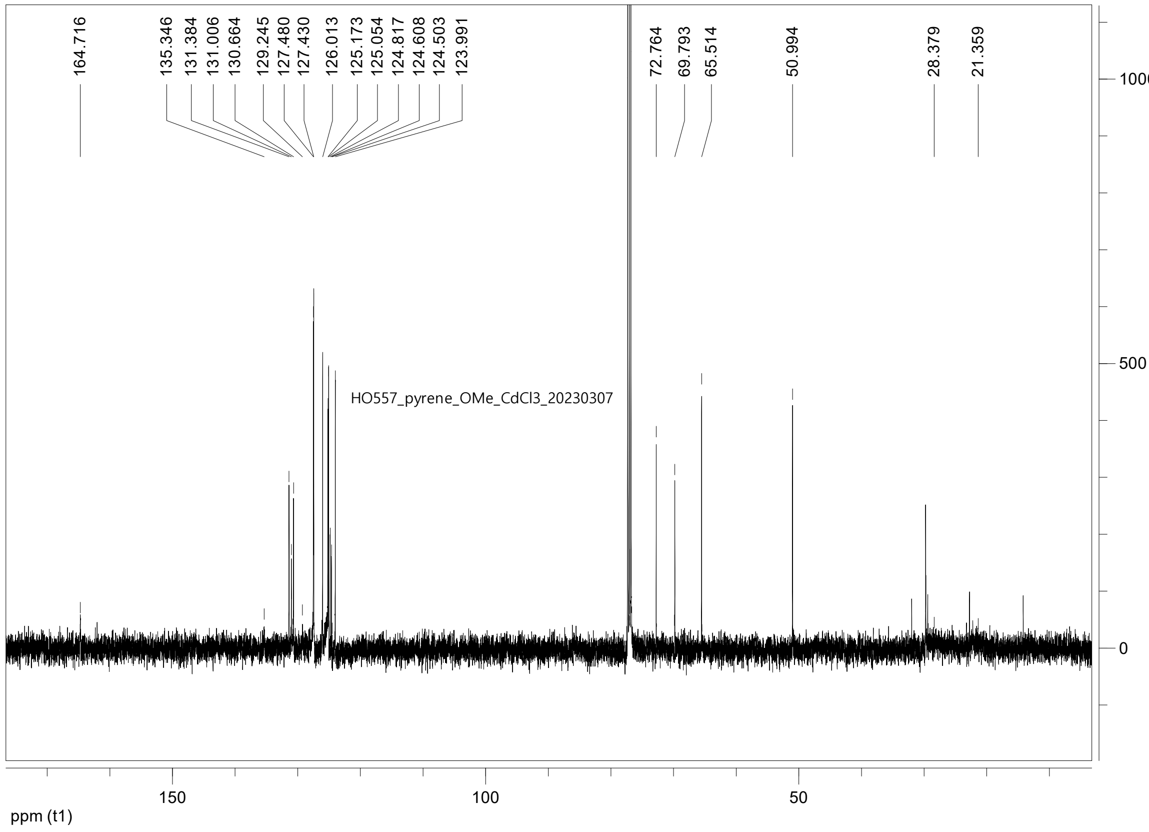

Figure S2: The ^{1}H NMR and ^{13}C NMR of compound **5**.

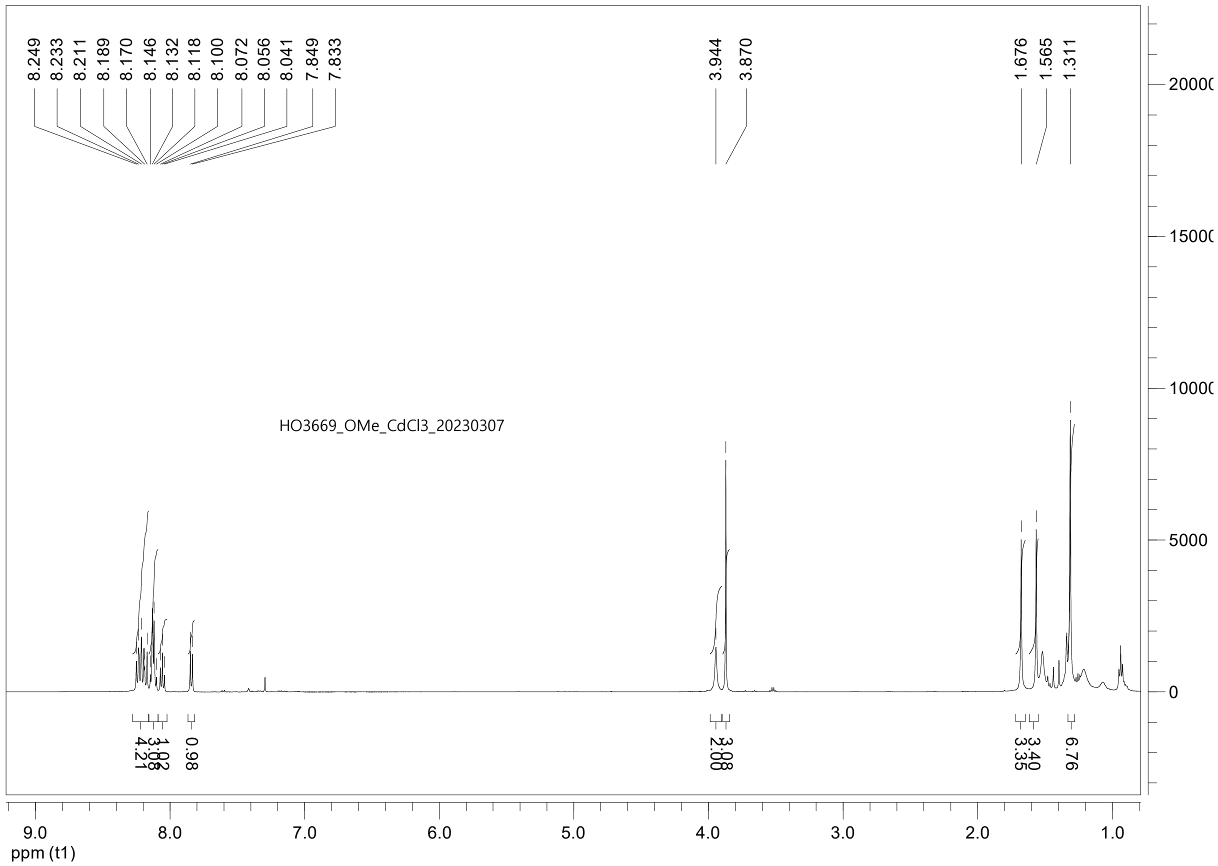

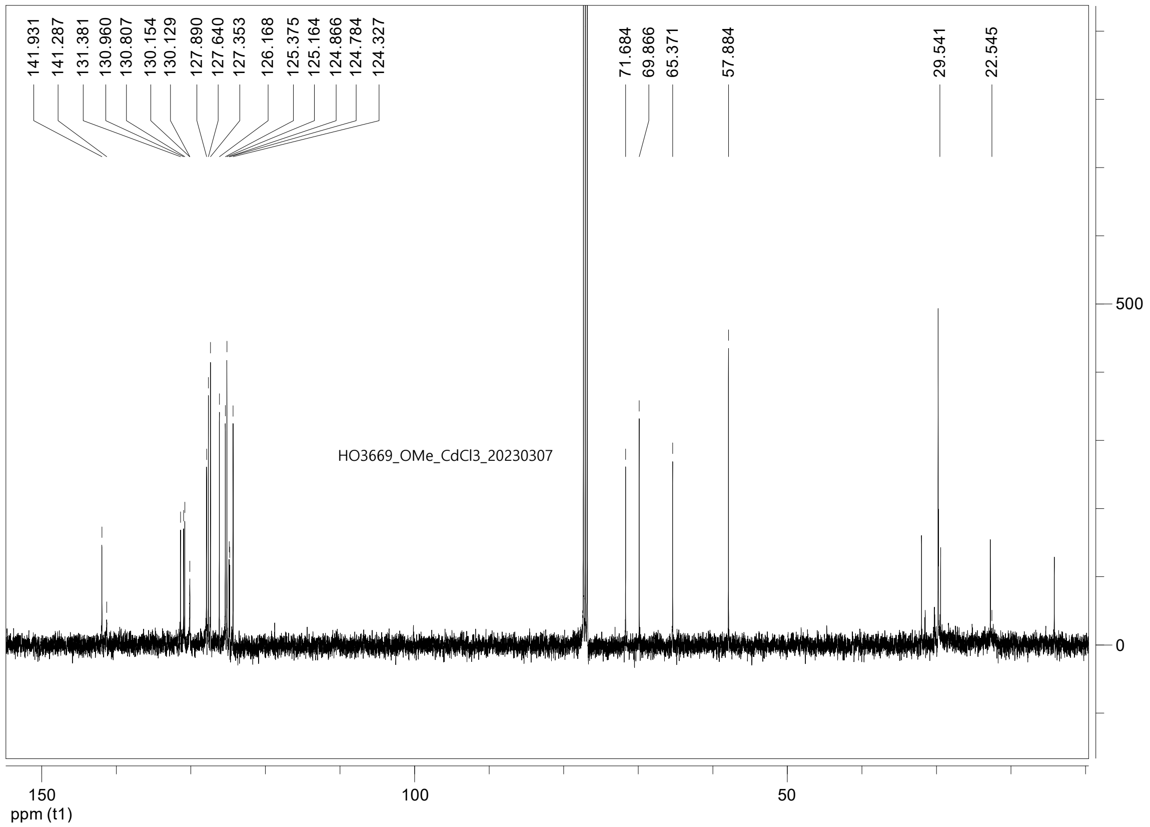

Figure S3: The ^{1}H NMR and ^{13}C NMR of compound **6**.
